# The nucleolus, an ally, and an enemy of cancer cells

**DOI:** 10.1007/s00418-018-1706-5

**Published:** 2018-08-13

**Authors:** Dariusz Stępiński

**Affiliations:** Department of Cytophysiology, Faculty of Biology and Environmental Protection, University of Łódź, Pomorska 141/143, 90-236 Łódź, Poland

**Keywords:** Nucleolus, Nucleolar and ribosomal proteins, Cell transformation, Nucleolar pro-cancer activity, Nucleolar anticancer strategies, Nucleolus-targeted therapy

## Abstract

The rates of ribosome production by a nucleolus and of protein biosynthesis by ribosomes are tightly correlated with the rate of cell growth and proliferation. All these processes must be matched and appropriately regulated to provide optimal cell functioning. Deregulation of certain factors, including oncogenes, controlling these processes, especially ribosome biosynthesis, can lead to cell transformation. Cancer cells are characterized by intense ribosome biosynthesis which is advantageous for their growth and proliferation. On the other hand, this feature can be engaged as an anticancer strategy. Numerous nucleolar factors such as nucleolar and ribosomal proteins as well as different RNAs, in addition to their role in ribosome biosynthesis, have other functions, including those associated with cancer biology. Some of them can contribute to cell transformation and cancer development. Others, under stress evoked by different factors which often hamper function of nucleoli and thus induce nucleolar/ribosomal stress, can participate in combating cancer cells. In this sense, intentional application of therapeutic agents affecting ribosome biosynthesis can cause either release of these molecules from nucleoli or their de novo biosynthesis to mediate the activation of pathways leading to elimination of harmful cells. This review underlines the role of a nucleolus not only as a ribosome constituting apparatus but also as a hub of both positive and negative control of cancer development. The article is mainly based on original papers concerning mechanisms in which the nucleolus is implicated directly or indirectly in processes associated with neoplasia.

## Introduction

Nucleoli are present in all eukaryotic cells with exception of those that have lost their nuclei during differentiation. Their main role, ribosome production, seems to be conservative in animals, plants, and yeasts. This process includes transcription of ribosomal DNA (rDNA), maturation of ribosomal RNA primary transcript (pre-rRNA), as well as assembly of ribosomal subunits and their transport to the cytoplasm (Olson and Dundr 2015; Raška et al. 2004; Stępiński 2014). In addition, they participate in many important cellular functions, including those responsible for health and disease (Núñez Villacís et al. 2018), and stress response and development. This concerns both animals (Lindström et al. 2018; Tsai and Pederson 2014; Tsekrekou et al. 2017) and plants (Kalinina et al. 2018; Ohbayashi and Sugiyama 2018).

Proliferating cells, including intensely dividing cancer cells, must meet the requirement of appropriate mass and size before they divide which is associated with huge demand for proteins. Nucleoli have to supply great enough number of translational nanomachineries, ribosomes, to provide optimal efficiency of protein biosynthesis. Thus, nucleoli perform a key function in maintenance of homeostasis in cells, and they can directly influence cell cycle progression, cell growth, and proliferation (Mayer and Grummt 2006; Zhou et al. 2018a). Interestingly, this concerns also plants in which certain nucleolar proteins are preferentially involved in promotion of leaf cell proliferation (Kojima et al. 2018).

Nucleoli synthesize half of all cell transcript pool and produce ca. 2 million ribosomes during 15 h. To complete this task, a proliferating cell utilizes up to 80% of energetic and material resources (Schmidt 1999). Hence, ribosome biosynthesis must be precisely controlled and adjusted to cell needs to ensure rational management of these resources. For example, Sfp1 and Sch9, factors that regulate processes depending on accessibility of nutrients and probably delay cell cycle until cells reach right sizes, make close correlation between cell growth and ribosome biosynthesis possible. Although this refers to yeast cells, analogs of these factors were identified in animal cells (Lempiäinen and Shore 2009; Rudra and Warner 2004).

A relationship between morphology and function of nucleoli and certain features of dividing cells was observed long time ago. The nucleolar size was one of the first parameters by means of which the intensity of cell growth and proliferation, mostly cancer cells, was estimated. Hypertrophy of nucleoli, manifested by their increase in size and number, is correlated with enhanced rate of cell proliferation and growth in cancer tissues (Donizy et al. 2017). A positive correlation between nucleolar size and intensity of ribosome biosynthesis was quite well proven in cells undergoing transformation as well as during further cancer development. Consistently, the enlarged nucleoli and increased cell proliferation are observed along with more intensive rDNA transcriptional activity which is frequently accompanied by enhanced expression of such factors involved in various stages of ribosome biosynthesis as upstream-binding factor (UBF), DNA topoisomerase I, fibrillarin, argyrophylic proteins (AgNOR proteins), including nucleolin (NCL) and nucleophosmin (NPM, B23), as well as small nucleolar RNAs (snoRNAs) and ribosomal proteins (RPs) of both small (RPS) and large (RPL) ribosomal subunits (Chang et al. 2016; Derenzini et al. 1998). However, big nucleoli are not exclusively characteristics of intensively proliferating cells producing ribosomes as enlarged nucleoli are also present in apoptotic leukemic granulocytic progenitors (Smetana et al. 2017) and in chilling-stressed plant cells (Stępiński 2009) and both show reduced nucleolar activity. It has also been suggested that increased aggressiveness of cancer cells is associated not only with augmented rRNA biosynthesis but also with activation of specific pathways of pre-rRNA maturation during which new post-transcriptional modifications are introduced into rRNA leading to increase in biosynthesis of ribosomes and to changes of their translational functionality (Belin et al. 2009).

Since ribosome biosynthesis is a driving force for cancer cells, they are more susceptible to disruption of ribosome production than normal cells, and this susceptibility is used to combat them (Bywater et al. 2012). A growing body of evidence suggests that disorder of nucleoli is a conservative mechanism leading to developmental cell death at least in certain cell types of multicellular organisms, including animals and plants, as well as in eukaryotic protist *Dictyostelium* (Golstein 2017; Luciani et al. 2016). In this context, natural or intentional disruption of nucleolar morphology and/or functioning can provoke death of eukaryotic cells, including cancer ones.

**Fig. 1:**
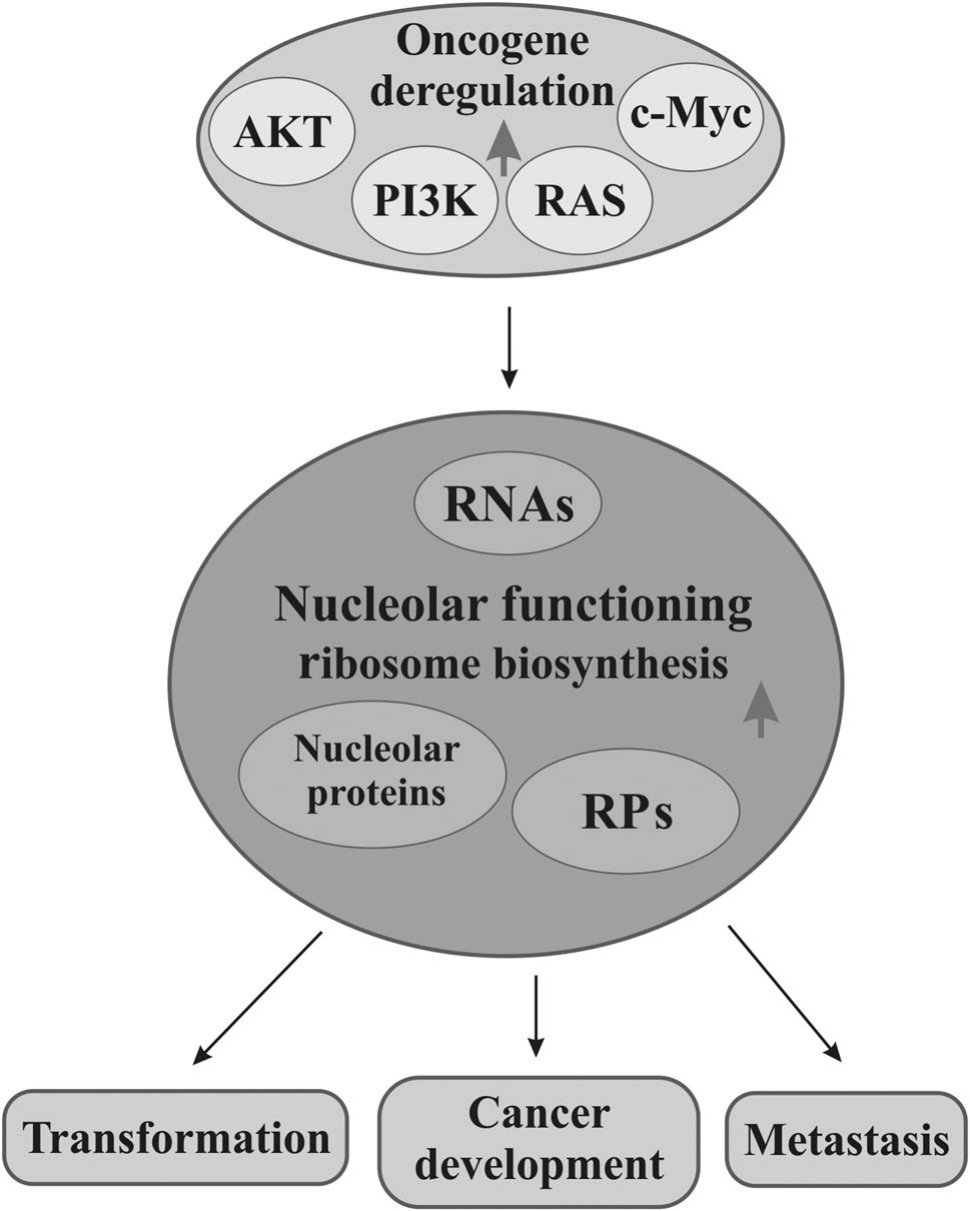
Cancerous processes associated with nucleolar functioning. Deregulation, especially overexpression of such oncogenes as AKT, PI3K, Ras, or c-Myc, causes upregulation of ribosomal (RPs) and nucleolar proteins which translates to more intensive nucleolar functioning (gray arrow). Consequently, increased ribosome biosynthesis drives processes related to neoplasia, i.e., cell transformation, cancer development, or metastasis

**Fig. 2:**
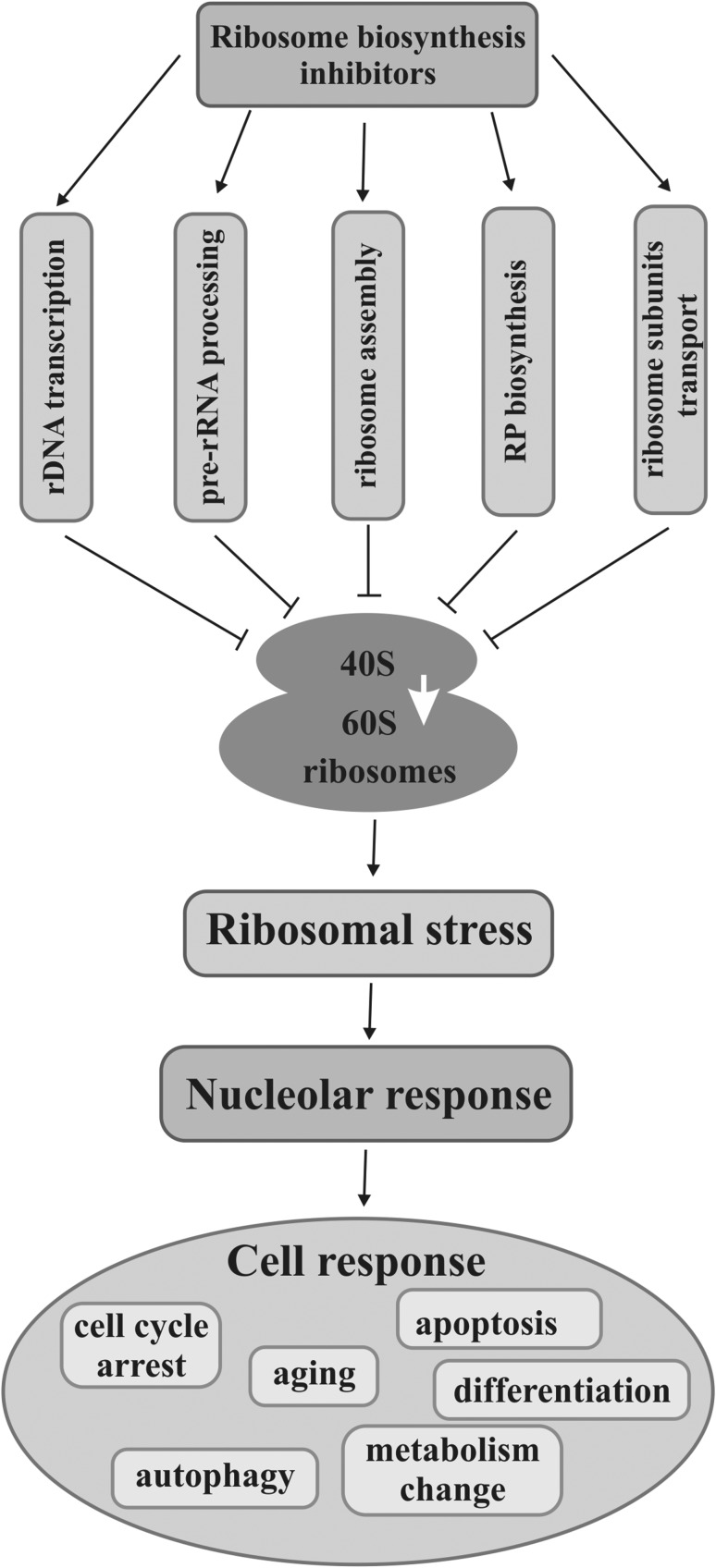
Disruption of ribosome biosynthesis can stop cancer cell development or even kill them. Inhibition of any stage of ribosome biosynthesis, i.e., rDNA transcription, pre-rRNA processing, ribosome assembly, RP biosynthesis, or transport of ribosomal particles with chemical or physical agents results in reduction of ribosome production which elicits nucleolar/ribosomal stress. Nucleolus responds to the stress by releasing nucleolar factors that mediate activation of pathways leading cells to the specific destinations such as cell cycle arrest, aging, autophagy, apoptosis, and cell differentiation, or to metabolism change

Carcinogenesis and cancer development in humans are often associated with increased activity of oncogenes on one hand and inactivation of suppressors on the other. A lot of protooncogenic factors such as AKT (protein kinase B), PI3K (phosphatidylinositol 3-kinase), Ras (a family of small GTP binding proteins), and c-Myc (a family of regulator gens and protooncogenes coding for transcriptional factors) take part in the regulation of various stages of ribosome biosynthesis in normal cells, whereas their deregulation leads to intensified ribosome production which may contribute to tumorigenesis (Devlin et al. 2013; Sriskanthadevan-Pirahas et al. 2018). Moreover, many other proteins or different RNAs, which are related to ribosome production, perform non-ribosomal functions in a nucleolus or are just sequestrated in it, can be engaged in malignancy, including transformation, cancer development, and metastasis. In this context, a nucleolus favors neoplasia (Fig. 1). On the other hand, a nucleolus can have anticancer activity when ribosome biosynthesis is impaired by any stressor, including intentional therapeutic action, which induces a nucleolar/ribosomal stress followed by a protective response. In this case, a nucleolus can mediate activation of pathways with or without p53, a suppressor transcriptional factor, by means of nucleolar or ribosomal proteins (Fig. 2).

As a number of discovered nucleolar factors still grow as well as new functions of well-known nucleolar factors are revealed in relation to cancer biology, this review summarizes the previous and latest knowledge concerning this issue.

## A nucleolus as a support of cancer cells

**Fig. 3:**
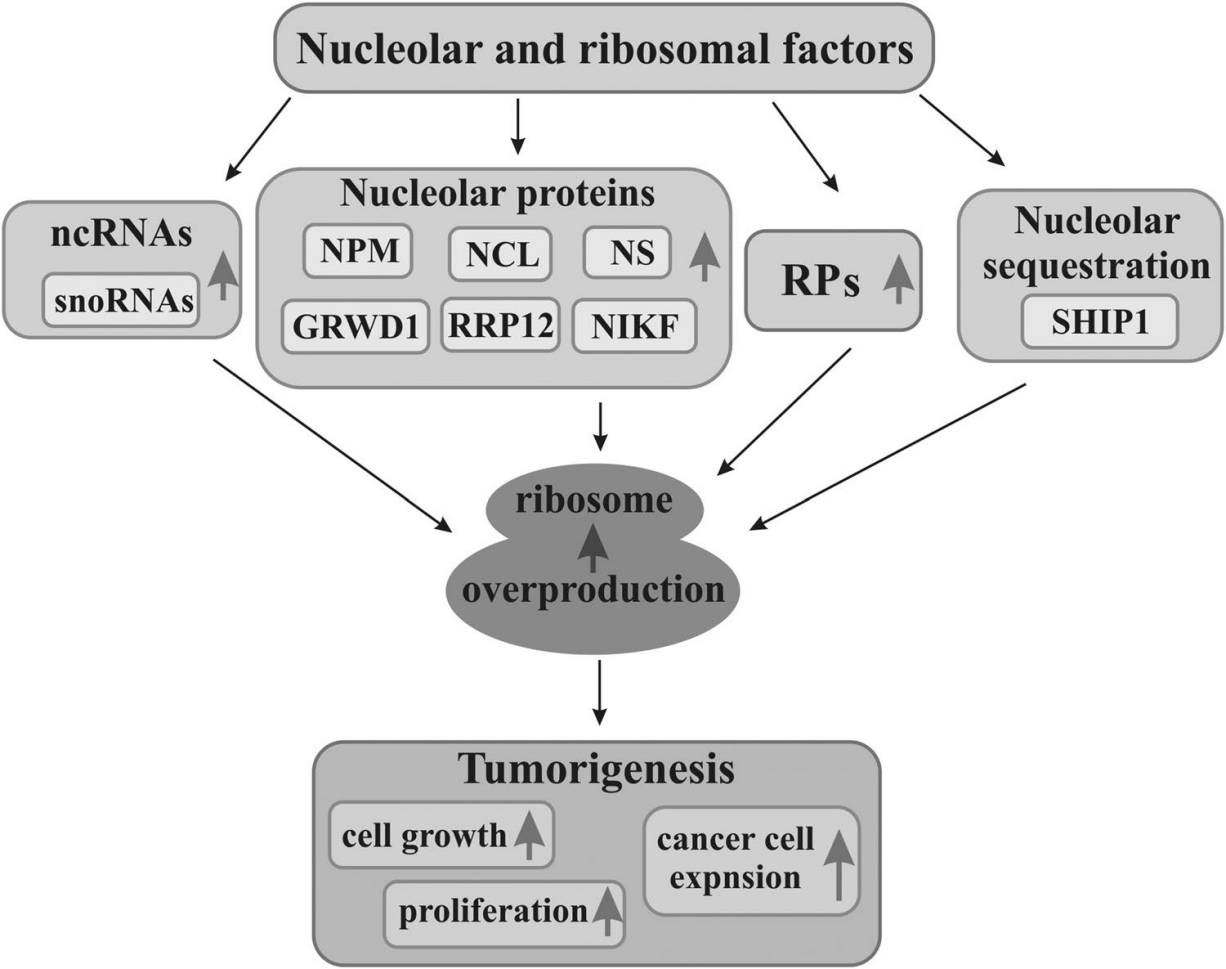
Nucleolar factors promoting cancer development. Numerous factors functionally connected with nucleolus such as non-coding RNAs (ncRNAs), nucleolar proteins, including nucleophosmin (NPM), nucleolin (NCL), nucleostemin (NS), glutamate-rich WD40 repeat containing 1 (GRWD1), ribosomal RNA processing 12 (RRP12), nucleolar protein interacting with the forkhead-associated (FHA) domain of pKi-67 (NIKF), and some ribosomal proteins (RPs) which are overexpressed (gray arrows), as well as factors sequestrated by nucleoli, including SHIP1, enhance ribosome biosynthesis (gray arrow). Overproduction of ribosomes makes cell growth, proliferation, and cancer cell expansion (gray arrow) more intensive, thereby enhancing tumorigenesis

The intensity of ribosome production translates to the efficiency of protein biosynthesis. Both these processes play essential roles in growth and proliferation of eukaryotic cells which are generally thought to be critical for tumorigenesis and cancer development (Bastide and David 2018; Bustelo and Dosil 2018). Impairment of ribosome biosynthesis considerably influences these processes, and thus, the mechanism coordinating growth and cell cycle with ribosome production must function efficiently. Mammalian cells quickly adjust the rate of ribosome production depending on availability of material and energetic resources and on the mitogenic factors promoting cell growth and divisions. In this regard, cancer cells seem to be privileged, and thus, their abnormal rapid growth and proliferation occur in contrast to differentiated or quiescent cells which lost ability to divide or even to proliferating but normal cells. Hence, intensified or reduced ribosome biosynthesis drives and restrains cell growth and proliferation, respectively. Especially increased rDNA transcription and enhanced expression of key factors involved in ribosome biosynthesis favor cell transformation, whereas hyperproduction of ribosomes promotes cancer expansion (Fig. 3) (Chang et al. 2016; Derenzini et al. 2017). It was shown that although 45S rDNA is lost during intense replication in rapidly proliferating cancer cells, amplification of 5S rDNA, occurring in these cells, stimulates proliferation, nucleolar activity, and ribosome production (Wang and Lemos 2017).

Ribosome biosynthesis is a complex process which can be controlled and affected at many stages, including rDNA transcription, maturation of pre-rRNA, RP biosynthesis, as well as ribosomal subunit assembly and their transport. All factors that positively regulate ribosome biosynthesis at any stage simultaneously promote cell growth and proliferation. Their deregulation towards much more intensive ribosome biosynthesis can lead to enhanced proliferation and tumorigenesis (Fig. 3). On the other hand, disturbed functioning of these factors reducing ribosome production can activate pathways leading to the halt of cell divisions (Ghalei et al. 2015; Peng et al. 2010; Yuan et al. 2018). Ribosome formation involves coordinated action of three main RNA polymerases, I, II, and III, so that adequate amounts of rRNA transcripts, RPs, and factors necessary for this process could be synthesized. Hence, ribosome biosynthesis needs tight regulation (Lempiäinen and Shore 2009). mTORC1 (mammalian/mechanistic target of rapamycin complex 1) pathway coordinates action of these three polymerases and adjusts biosynthesis of proteins, including RPs, and ribosome manufacture to environmental conditions, nutritional resources, and energy produced by mitochondria in mammalian cells under physiological conditions (Fyfe et al. 2018; Vizoso-Vázquez et al. 2018). Increased activity of this pathway is the most probable cause of many diseases, including tumorigenesis and development of great number of different cancer types (Hannan et al. 2011; Mayer and Grummt 2006; Morita et al. 2015). Furthermore, mTORC1/C2 is able to enact transcriptional and translational reprogramming during DNA damage stress to survive cancer cells (Silvera et al. 2017).

The oncogenes seem to be important factors coordinating ribosome biosynthesis, cell growth, and proliferation. Their optimal level ensures an appropriate course of these processes, whereas their deregulation may increase biosynthetic and proliferative activities, characteristic of transformed cells. c-Myc is one of the main protooncogenic transcriptional factors which enhances growth, proliferation, and metabolism, thereby promoting cancer development through different mechanisms (Pan et al. 2018). It can regulate activity of genes connected with energetic metabolism, protein biosynthesis, and ribosome production (van Riggelen et al. 2010). Constitutive c-Myc level ensures homeostasis and optimal functioning of normal cells and ultimately mammal development. When c-Myc is upregulated, it usually enhances transcription and ribosome production, and, thus, drives cells towards transformation and cancer development. Therefore, it is not astonishing that its overexpression is observed in 15–20% of different types of human cancers (Adhikary and Eilers 2005; Lewis et al. 2018; Nesbit et al. 1999; Niemas-Teshiba et al. 2018; Pelengaris et al. 2002; Schmidt 1999). Given that c-Myc regulates general transcription in a cell and that rRNA transcripts are most abundant among all cell transcripts, it is supposed that c-Myc substantially controls rRNA biosynthesis. Indeed, c-Myc is able to regulate ribosome biosynthesis at many levels, including rDNA transcription in nucleolus where it directly recruits RNA polymerase I and activates rDNA transcription as well as RP biosynthesis (Arabi et al. 2005), while cells with muted c-Myc activity show significantly lower efficacy of overall transcription and reduced growth, colonization, cell cycle progression, and capability of carcinogenesis as a result of decreased expression of c-Myc-regulated genes that control these processes. In general, permanently high c-Myc activity is advantageous for cancer development, whereas its inactivation results in cancer suppression, and thus, elimination of c-Myc activity can be a potential method in anticancer therapy (Niu et al. 2015). Interestingly, there is a mechanism that may act in the opposite direction, when specific inhibition of hyperactive rDNA transcription and protein biosynthesis can suppress MYC/MYCN protein expression resulting in reduced neuroblastoma growth (Niemas-Teshiba et al. 2018).

Moreover, c-Myc cooperates with AKT which, similarly as c-Myc, is a key factor mediating the regulation of cell growth and proliferation through the control of various stages of ribosome biosynthesis in normal cells. AKT influences these processes acting in PI3K-AKT-mTORC1 pathway and independently of this axis (Chan et al. 2011). In the latter case, AKT enhances rDNA transcription and, in consequence, ribosome production through phosphorylation of casein kinase II (CK2). It in turn phosphorylates the transcription initiation factor I (TIFI-A), an essential element of initiation of rRNA gene transcription, thereby activates it. This phosphorylation ensures its (1) stabilization due to prevention against its ubiquitination and then degradation in proteasomes, (2) translocation from nucleoplasm to a nucleolus, and (3) increased effectiveness of cooperation with RNA polymerase I (Nguyen and Mitchell 2013). AKT–CK2 interaction was proved to play pivotal role in the regulation of cell proliferation and transformation (Ponce et al. 2011). Furthermore, deregulation of the AKT signaling pathway consisting in its hyperactivation or mutations of suppressor genes (e.g. PTEN, phosphatase and tensin homolog protein) results in the activation of the processes which are symptoms of neoplasia (Hannan et al. 2011). Although it seems that c-Myc and AKT act in separate pathways, they complement each other to have a synergistic effect on ribosome biosynthesis and to provide intense growth of cancer cells. That is why, AKT is indispensable to ensure the maximal activity of c-Myc in malignant cancers, whereas inactivation of AKT significantly reduces ribosome production (Chan et al. 2011) and probably diminishes risk of transformation. In addition, AKT participates in other signaling pathways favoring cancers (Jia et al. 2018; Zhou et al. 2018b). Thus, targeting AKT-dependent pathways leading to cell cycle arrest, autophagy, or apoptosis may be used as an anticancer strategy (Lin et al. 2018b; Xu et al. 2018; Yang et al. 2018).

Numerous nucleolar proteins are recognized to take part in cell growth and proliferation through their direct or indirect impact i.a. on ribosome biosynthesis regulation. Novel functions of nucleolar proteins have been recently added to their well-known roles in ribosome production. Growing number of nucleolar proteins and different RNAs associated with a nucleolus, whose action is related to cancer processes, are being identified (Fig. 3).

Nucleophosmin (B23, NPM) is a multifunctional protein closely related to cell growth and proliferation. Under certain circumstances, NPM can trigger tumorigenic processes and be a potential oncogene even if NPM can activate PI3K/AKT pathway (Chen et al. 2018); under others, it can act as a suppressor. In many human cancer types, NPM undergoes deletion, translocation, and mutation. The latter frequently occurs in acute myeloid leukemia (AML) (Nabbouh et al. 2017). Cancers are also accompanied by NPM deregulation, and up- and downregulation. However, expression level of NPM and its cancerogenic function are not unequivocal and can differ in many types of cancers depending on a cell type, genetic context, or micro-environmental stimuli. Interestingly, expression of NPM at a transcription level can be converse to its expression at a translation level in the same type of cancer (Bonetti et al. 2008; Box et al. 2016; Leal et al. 2014). NPM is overexpressed in many cancers, especially in highly malignant tumor subtypes (Li et al. 2017; Sawazaki et al. 2017). Hyperexpression of NPM, both of its transcripts and proteins, was observed i.a. in the adenomatous tissue and colorectal cancer (CRC). It has been suggested that this overexpression plays a critical role mainly during the initial stages of CRC development (Wong et al. 2013). Although NPM resides predominantly in a nucleolus, where it participates in ribosome biogenesis, it can also function in extranucleolar nucleoplasm where it controls c-Myc activity. NPM overexpression significantly induces c-Myc oncogenic action and influences expression of the c-Myc-regulated genes resulting in enhanced cell proliferation and transformation, while NPM overexpression alone, with no c-Myc participation, has rather a little effect on these processes (Li et al. 2008). There is also a mechanism acting in the other direction in which c-Myc mediates overexpression of NPM and ribosomal proteins in NOTCH1-mutated chronic lymphocytic leukemia cells (Pozzo et al. 2017). Moreover, upregulated NPM is disadvantageous from therapeutic point of view, because it contributes to multidrug resistance in breast carcinoma (Chen et al. 2018). Notwithstanding, usually lowered NPM expression which is observed in gastric cancer may be the reason of gastric carcinogenesis (Leal et al. 2014). On the other hand, its decreased expression can arrest cell cycle and cause early cell aging through p53 pathway (Wong et al. 2013). NPM may also influence cancers through oxidative stress homeostasis as NPM regulates the expression of peroxiredoxin 6, a member of antioxidant protein family, in tumor cells (Liu et al. 2017a). In addition to cancer involvement, NPM is also implicated in some viral infections (Lobaina and Perera 2018).

Nucleolin (NCL, C23) is a phosphoprotein with a lot of cellular roles, including oncofunction. NCL because of its pro-tumorigenic activity is implicated in many mechanisms being direct or indirect chain link of various pathways characteristic of cancer development (Liao et al. 2018; Yu et al. 2018). NCL phosphorylated by CK2 is involved in cell proliferation, whereas mutated form of NCL, unable to be phosphorylated, reduces cell vitality and proliferation, and probably directs cells to apoptosis through p53 pathway (Xiao et al. 2014). Since NCL overexpression promotes cell proliferation and transformation, its increased level is observed in many human tumor types (Berger et al. 2015). This is the case in the vast majority of patients with pancreatic cancer in which NCL level is positively correlated with proliferative activity of pancreatic cells, their invasion, and metastasis. In addition, nucleolin displays specific expression and localization in various cancers as it is in the case of circulating prostate cancer cells, so this feature can be helpful in their identification (Chalfin et al. 2017). Although NCL is one of the main nucleolar proteins, it also functions in nucleoplasm and cytoplasm. Moreover, surface NCL, which is anchored in the plasma membrane of hepatoma cells, interacts with the hepatoma-derived growth factor and activates pro-oncogenic PI3K/Akt pathway leading to liver carcinogenesis in humans, while blocked access to membrane NCL impairs activity of this pathway and reduces the oncogenic potential of hepatoma cells (Chen et al. 2015). Furthermore, NCL cooperates with ErbB2, a member of the receptor tyrosine kinase family, which takes part in a signaling pathway that can elicit oncogenic effects, i.e., increased cell growth, proliferation, migration, and survival. Although this cooperation occurs in normal human cells and has oncogenic potential, it activates pathways with the aid of other oncogenes resulting in the enhanced tumorigenicity and in the invasion of breast carcinoma cells when both proteins are overexpressed. Hence, in patients in which NCL overexpression activates ErbB2, the increased risk of breast cancer and the reduced survival are observed, whereas specific inhibition of NCL expression by GroA (AS1411) or shRNA transfection prevents NCL/ErbB2 interaction activating oncogenic pathways in ErbB2-positive breast cancer cells, thereby reduces oncogenic transformation and limits tumor growth and disease progression (Wolfson et al. 2016, 2018). Interestingly, NCL can act as a suppressor in intensively proliferating cells with heightened level of NCL through induction of the mechanisms stabilizing p53 which, in consequence, activates p53-dependent pathways and leads to the reduced cell growth (Saxena et al. 2006; Xiao et al. 2014). As the role of NCL in tumorigenesis of certain cancers is crucial, it could be a target protein for anticancer agents. A natural product, curcumol, exerts biological anticancer effects on nasopharyngeal carcinoma cells by NCL inhibition and it operates with minimal adverse effects (Wang et al. 2018).

Nucleostemin (NS) is a protein detected in nucleoli of intensively proliferating cells, mainly stem cells and of several types of cancer cells, whereas its expression decreases in differentiating cells and disappears in fully differentiated cells. Hence, NS is thought to perform an important function in the control of cell proliferation (Tsai and McKay 2002). Indeed, NS silencing reduces proliferation, induces differentiation, and eventually drives acute promyelocytic leukemia cells to autophagy (Fakhimahmadi et al. 2017). Similarly, the crucial role of NS in functioning of cancer cells is witnessed by the fact that the NS-deficient ovarian cancer cells are led to cell cycle arrest and to an increase in apoptosis. In addition, the tumorigenic ability, growth rate, migration, and invasion are drastically inhibited in these cells (Wang et al. 2017a). Metastasis of gastric cancer cells as well as hepatocellular carcinoma are correlated with high expression of NS; moreover, patients with such expression have much shorter survival than those with low expression, while NS-knocked down cells are characterized by the reduction of proliferation and viability (Hua et al. 2017; Wu et al. 2015). NS influences the viability of proliferating cells, also because it protects the integrity of a replicating genome. Different outcomes of an interplay between statuses of NS and of p53 are observed both in normal and cancer cells. Furthermore, once nucleolar disintegration elicits nucleolar stress response consisting in the release of a large amount of NS from a nucleolus which stabilizes murine double minute 2 (Mdm2, an oncoprotein with ubiquitin ligase activity) in the nucleoplasm, p53-dependent pathways are activated. Thus, treatment with any agent that could disturb nucleolar integrity and release NS from a nucleolus or that could diminish NS expression would guide cells to final fates in the therapy of various cancer types (Hua et al. 2017; Tsai 2015; Wang et al. 2017a).

It was revealed that nucleolar protein glutamate-rich WD40 repeat containing 1 (GRWD1), implicated in ribosome biogenesis and other cellular functions, is a novel negative regulator of p53, and thus, it acts as a potential oncogene. GRWD1 upon nucleolar stress migrates to nucleoplasm where it interacts with RPL11 and prevents blocking of Mdm2 activity. In consequence, p53 is degraded and the cell lacks anticancer protection in the form of p53-induced pathways (Kayama et al. 2017). GRWD1 overexpression considerably reduces p53 activation and accelerates malignant transformation which correlates with poor prognosis for patients suffering from several cancers (Takafuji et al. 2017).

Nucleolar protein ribosomal RNA processing 12 (RRP12), engaged in ribosomal subunit maturation and export, seems to be crucial for regulation of p53 activity in osteosarcoma cells during nucleolar stress. When nucleolar function and structure are disrupted by actinomycin D (Act D) or doxorubicin (Dox), p53 pathways are activated; thereby, cell cycle arrest and apoptosis appear. According to this mechanism, RRP12 overexpression disrupts p53 stability and promotes cell resistance to cytotoxic stress, while RRP12 silencing enhances p53 activity and cell death. Thus, targeting RRP12 function in combination with the nucleolus-disintegrating agents could increase the efficacy of anticancer therapy (Choi et al. 2016).

Nucleolar protein interacting with the forkhead-associated (FHA) domain of pKi-67 (NIKF) is a protein residing both in cytoplasm and in a nucleolus where it is involved in rRNA maturation (Pan et al. 2015). Furthermore, it is involved in Ki-67-dependent cancer processes such as proliferation, migration, invasion, and metastasis. As Ki-67 is expressed solely in proliferating cells, also in those of many cancer types, including lung cancer, it serves as a diagnostic biomarker. NIKF expression promotes these processes through negative regulation of RUNX1, a transcription factor of casein kinase 1α (CK1α) which suppresses TCF4/β-catenin signaling. TCF4/β-catenin is a pro-metastatic pathway in which oncogenic β-catenin is a substrate for CK1α; thereby, it activates, among others, c-Myc. In the p53-deficient lung cancer cells, NIFK-CK1α-β-catenin axis increases considerably its pro-tumorigenic role (Lin et al. 2016a).

Special attention should be paid to RPs which also play other functions than that of ribosome-building elements not only in animals but also in plants (Enganti et al. 2018; Li et al. 2018). RPs can be the source of many diseases. Especially, mutations and deletions of RPs, both homo- and heterozygous, increase susceptibility to various diseases and trigger the development of some illnesses, including cancers (Hofman et al. 2017; Kazerounian et al. 2016). It turns out that hemizygous RP gene deletions are common and occur in almost half of cancers. Such cancer cells are particularly vulnerable to p53 action; thereby, tumor growth is inhibited via p53-dependent pathways in contrast to p53-mutated tumors (Ajore et al. 2017). However, correct RPs can also contribute to carcinogenesis (Ebert et al. 2008; Song et al. 2010). Interestingly, about a quarter of human RPs shows tissue-specific expression in cells of many normal and tumor tissues. This plasticity of RP expression is probably regulated by transcriptional factors (Guimaraes and Zavolan 2016). Furthermore, RP transcript expression, that is independent of protein expression, differs between normal and cancerous tissues, so it could be a novel method of tumor identification (Dolezal et al. 2018). Deregulation of RP expression, particularly its upregulation, which usually goes well with enhanced cell proliferative activity, occurs in many types of cancers. Abnormal expression of some RPs preferentially exhibit pro-tumorigenic activity. For example, overexpression of RPL23 negatively regulates apoptosis in higher risk myelodysplastic syndrome (Qi et al. 2017). RPS15a, which is overexpressed in hepatocellular carcinoma, promotes proliferation and tumor angiogenesis, while its inhibition exerts opposite effects (Guo et al. 2018). Other RPs of small ribosomal subunits, such as RPS19, RPS 21, and RPS24, are also upregulated in prostate cancer and they could be potential biomarkers of malignancy in this cancer type (Arthurs et al. 2017). However, expressions of some RP genes are significantly downregulated in nasopharyngeal carcinoma cells (Sim et al. 2017). From all RPs, RPL34 seems to be deregulated most frequently in many types of human cancers. Enhanced expression of RPL34 contributes to the cancerous transformation and development of gastric cancer in humans, while RNAi-mediated inhibition of its expression significantly reduces cell proliferation and escalates apoptosis (Liu et al. 2015b). Similar RPL34 action was observed in the cells of non-small cell lung cancer (NSCLC) (Yang et al. 2016a) as well as of esophageal cancer (Fan et al. 2017). Likewise, RPL34 overexpression in osteosarcoma promotes proliferation of malignant cells. In this case, RPL34, whose transcription is regulated by c-Myc, cooperates with certain subunits of the important initiating translation factor (eIF3) which probably results in increased protein biosynthesis, including those promoting growth (Luo et al. 2016). In the pancreatic cancer (PC) cells, RPL34 is hyperexpressed most probably through hypomethylation of the promoter of a gene encoding this protein. Here, RPL34 promotes cell proliferation, colony formation, migration, invasion, and drug resistance of PC, whereas siRNA-mediated RPL34 knockdown reduces these processes and, consequently, decreases PC tumor growth and metastasis which is accompanied by the induction of apoptosis (Wei et al. 2016). In general, RPL34 and some other RPs are thought to promote development and progress of cancer cells by regulation of the mitogen-activated protein kinase (MAPK) increasing their proliferation and as well as their resistance to apoptosis.

Apart from nucleolus-associated proteins, also various kinds of non-coding RNAs (ncRNAs) are implicated in the regulation of processes connected with carcinogenesis. Small nucleolar RNAs (snoRNAs) until recently were seen as housekeeping ncRNAs necessary mainly for pre-rRNA processing. Now, it should not be surprising that they are involved in neoplasia as ribosome biogenesis itself drives this process. Hence, participation of some snoRNAs in transformation and cancer development, during which deregulation of their expression occurs, is tightly related to enhanced ribosome production and more efficient cell proliferation and invasion. U3 and U8 snoRNAs are upregulated in many cancer types, including breast cancer, whilst under U3 or U8 depletion, the tumorigenic potential of lung and breast tumors decreases considerably and p53-induced apoptotic pathways are activated (Langhendries et al. 2016). Likewise, SNORD78 overexpression enhances cell proliferation in tumorigenesis of NSCLC and tumor-initiating cells, whereas SNORD78 repression arrests cell divisions and invasion (Zheng et al. 2015).

Some snoRNAs, similarly as in the case of many nucleolar proteins or RPs, are implicated in certain human cancer types rather without connection to ribosome biosynthesis. Such snoRNAs as SNORD50A or SNORD50B directly bind to oncogenes, including K-Ras, blocking their activity; thereby, the pathways leading to carcinogenesis in which they act are repressed. Locus deletions of these snoRNAs enhance tumorigenesis, so they are observed in many human cancers (Siprashvili et al. 2016). SNORA55 overexpression is associated with activation of oncogenic pathways in prostate tissue which is a driving force in the development of prostate cancer (PtC), while SNORA55 silencing stops proliferation and metastatic potential of PtC cells (Crea et al. 2016). On the other hand, the lowered expression of SNORD47 is associated with tumorigenesis in glioblastoma, while its upregulated expression suppresses invasion of glioma cells and extends patient’s survival (Xu et al. 2017). Altered snoRNA and piRNA expression occurs in patients with lung adenocarcinoma; furthermore, this differentiated expression allows to discriminate smokers and non-smokers (Nogueira Jorge et al. 2017). Furthermore, some snoRNAs can regulate expression of other genes, including those coding for other ncRNAs which are involved in tumorigenesis (Krishnan et al. 2016; Su et al. 2015). Interestingly, human snoRNA-93 is processed into RNA derived from small nucleolar RNA, sdRNA-93, which enhances breast malignancy participating in miRNA-like regulation (Patterson et al. 2017). Therefore, snoRNAs can be considered as a new group of pro-oncogenic factors on one hand, while, on the other, they can act as an extra tool in tumor suppression. Application of blockers of snoRNA activity resulting in ribosome biosynthesis inhibition and eliciting nucleolar stress can activate p53-depended pathways. Moreover, increased or specific expression of particular snoRNAs in many human cancer types becomes an additional biomarker used in cancer recognition, progression estimation, prognosis, or even in evaluation of metastasis ability.

Moreover, deregulation of RPs and snoRNAs and their pro-oncogenic activity may result from abnormal expression of miRNA or from polymorphism of miRNA genes which control the expression of these and other essential factors involved in ribosome biosynthesis. For example, miRNA-7641 deregulates the expression of genes coding i.a. RPS16 and other RPs in breast cancer, whilst inhibition of miRNA-7641 upregulates expression of its target genes and sensitizes cancer cells to antitumor agents which enhances efficacy of therapy (Reza et al. 2017). Furthermore, genetic variants of some miRNAs, which upregulate rRNA maturation, RP, and snoRNA synthesis, contribute to ribosome hyperproduction and promote progression of endometriosis and its derivative carcinoma, while CX5461, a nucleolar stress elicitor, halts cell cycle and proliferation which leads cells towards apoptosis (Chang et al. 2016). Interestingly, over a dozen miRNA types, which derive from different transcribed regions of human rRNA gene, including ITS (internal transcribed spacer), ETS (external transcribed spacer) or 18S rRNA, termed rDNA-hosted pre-miRNA analogs (rmiRNAs) were identified. They can be formed during normal pre-rRNA processing or as a result of extra-processing of rRNA originating from ribosomes which are disintegrated. These rmiRNAs are suggested to participate in the control of genes implicated in the response to stress or of cancer-related genes (Yoshikawa and Fujii 2016). Furthermore, the expression of some miRNAs is useful in diagnostics of certain cancers, including pancreatic ductal adenocarcinoma. Some RNAs, including snoRNAs, especially U91, showing high stability in cancer tissues, are used in the normalization of quantitative estimation of miRNA expression in this cancer type (Popov et al. 2015).

In addition to the above-mentioned nucleolar factors that are directly involved in cancer processes, the regulatory function of a nucleolus due to its ability to sequestrate of different molecules, including those related to carcinogenesis, is well documented. Functional SHIP1 (inositol 5-phosphatase) regulates proliferation of hematopoietic cells negatively, thus it is accepted as a cancer suppressor, whereas its reduced expression or activity can lead to leukemogenesis, including chronic myeloid leukemia (CML) or AML. SHIP1 accumulates together with i.a. p53 in nucleolar vacuoles (nucleolar cavities) of both normal and leukemic hematopoietic cells. Nucleolar sequestration of SHIP1 is suggested to be the mechanism limiting its suppressive function, and thus, it may contribute to tumorigenesis (Ehm et al. 2015).

## A nucleolus as an opponent of cancer cells without p53 involvement

**Fig. 4:**
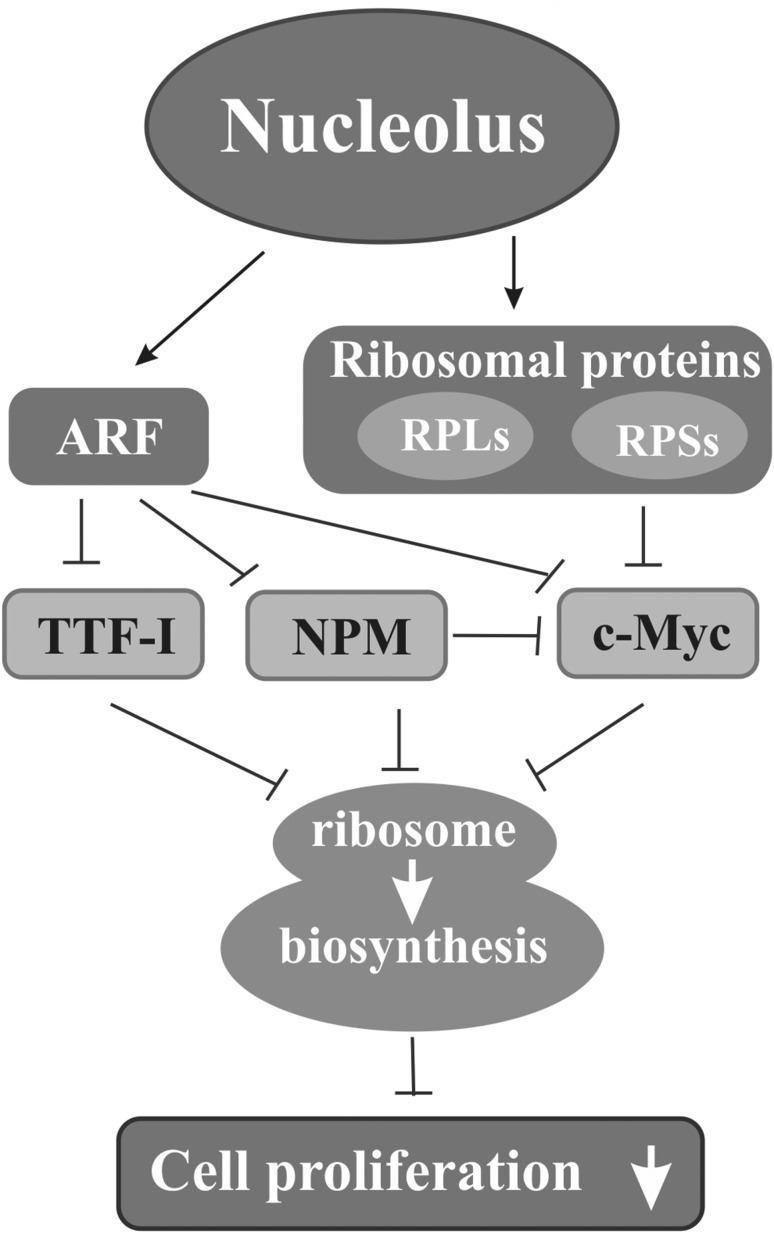
Nucleolar proteins in reduction of cancer development. Some nucleolar proteins such as ARF and certain ribosomal proteins (RPs) of small (RPSs) and of large (RPLs) ribosomal subunit can inhibit the activities of factors participating in and regulating ribosome biosynthesis such as i.a. transcription termination factor I (TTF-I), nucleophosmin (NPM), or c-Myc; in addition, NPM alone can inactivate c-Myc. This results in reduced or stopped ribosome production (white arrows) followed by inhibition of cell proliferation

Many proteins connected with a nucleolus, including typical nucleolar ones that are directly involved in ribosome biogenesis, RPs, as well as extra nucleolar proteins that indirectly influence ribosome production, may take part in the anticancer protection participating in the pathways activated to eliminate cancer cells. Some of these proteins are implicated in pathways activated by the main cellular suppressor, p53; others are involved in the p53-independent pathways, and several in both (Fig. 4).

**Fig. 5:**
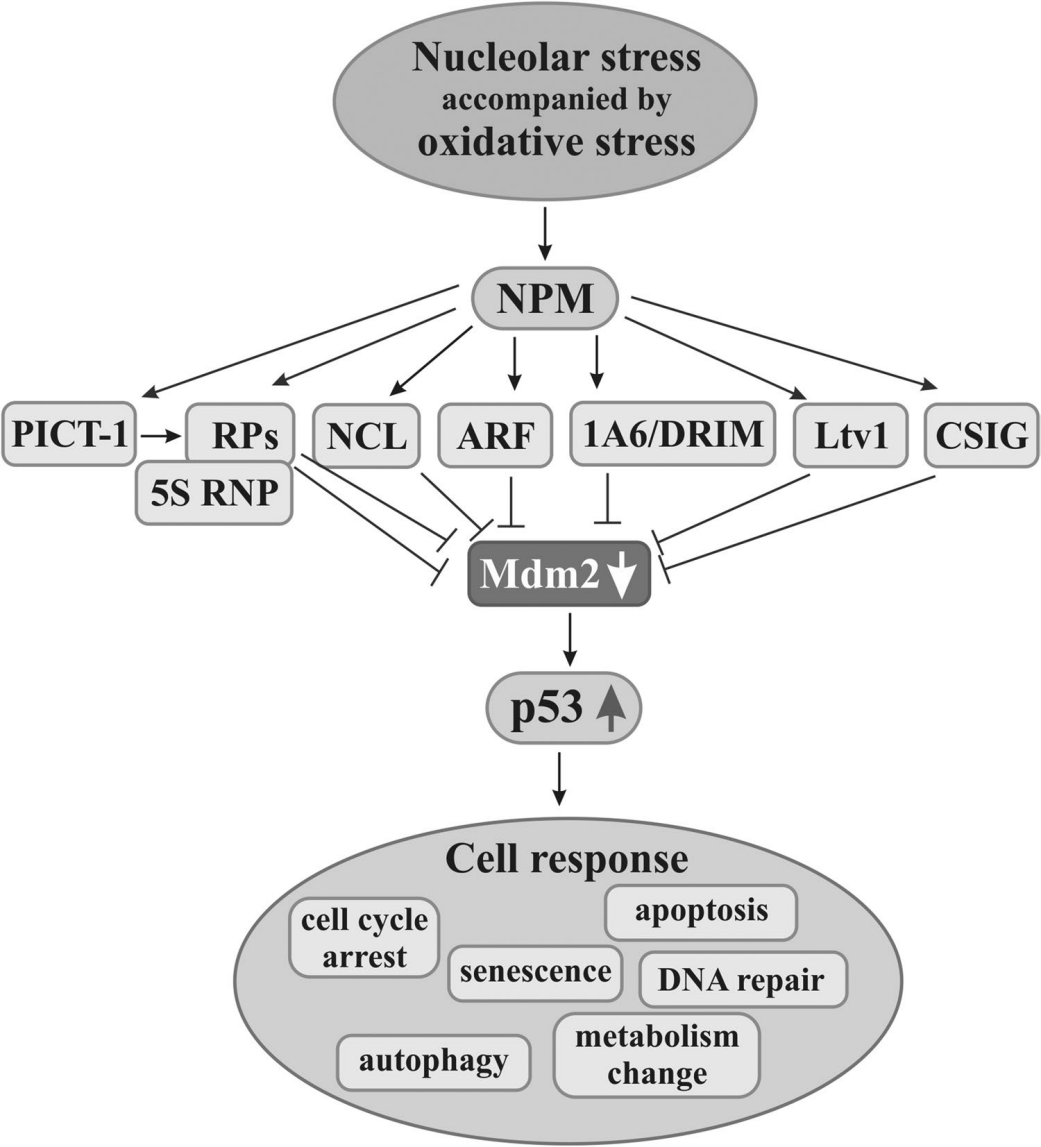
Nucleolar factors in the activation of p53-dependent anticancer pathways. Stress factors in addition to nucleolar stress usually also elicit oxidative one. Nucleophosmin (NPM) seems to be a nucleolar molecule which initiates nucleolar response by recognizing nucleolar stress through oxidative stress. Then, other nucleolar factors such as nucleolin (NCL), ARF, 1A6/DRIM, Ltv1, or CSIG can block activity of Mdm2 which, under normal conditions, reduces p53 level preventing activation of p53-dependent pathways. Moreover, PICT-1-induced process of Mdm2 activity impairment is mediated by ribosomal proteins (RPs) both by RPs alone and in complex with 5S RNP. Mdm2 inhibition causes increase in p53 level, its stabilization, and activation of p53-mediated pathways leading cells to apoptosis, autophagy, senescence, metabolism change, and cell cycle arrest, or to DNA repair

ARF (p14^ARF^ or p19^ARF^ in human or mouse, respectively) occurs mainly in a nucleolus, but it can also function in the nucleoplasm. ARF is a cancer suppressor, and hence, loss of its suppressor function appears in c.a. 40% of human cancers (Sharpless 2005). Disfunction of ARF may not be involved in tumor initiation, but it may promote tumor progression in some cases (Wang et al. 2017b). Antioncogenic activity of ARF depends on its proper expression, which may be modulated at many levels, including transcriptional regulation and post-translational modifications, while its deregulation promotes tumorigenesis (Ko et al. 2018). ARF is activated mostly in response to abnormally high levels of mitogenic signals due to protooncogene overexpression. It is involved in the anticancer protection via pathways with (Fig. 5) or without p53. In the latter case, ARF may bind to c-Myc only when its amount exceeds that needed for regular cell proliferation. c-Myc relocalization from the nucleolus to the nucleoplasm is observed in such a situation. Nucleolar sequestration of c-Myc prevents the activation of genes associated with cell cycle regulation which results in the inhibition of cell proliferation (Datta et al. 2004). In addition, ARF can indirectly halt proliferation through negative ribosome biosynthesis regulation. rDNA transcription may be disturbed by preventing RNA polymerase I-accompanied transcription termination factor I (TTF-I), a key rDNA transcriptional factor, from entering a nucleolus. TTF-I nucleolar localization is ensured by NPM which binds to a nucleolar localization sequence (NoLS) of TTF-I. This domain may be blocked by direct interaction of ARF with TTF-I outside a nucleolus which hinders TTF-I getting into a nucleolus. This results in blocking of both functioning of RNA polymerase I complex and co-transcriptional pre-rRNA processing (Lessard et al. 2010). It was noted earlier that ARF hampered the pre-rRNA maturation, delaying formation of the intermediate products during pre-rRNA processing, and thus, it handicapped ribosome production which inhibited cancer development via pathways without p53 (Sugimoto et al. 2003). ARF also negatively regulates activity of a nuclear factor, E2-related factor 2 (NRF2), an activator of genes connected with reactive oxygen species (ROS)-induced ferroptosis. Inhibition of NRF2 by ARF sensitizes cancer cells to ferroptosis and, thus, suppresses tumor growth in p53-independent manner in response to oxidative stress (Chen et al. 2017). However, in addition to its suppressive activity, ARF can also favor cancers as its expression contributes to resistance to chemotherapy in muscle-invasive bladder cancer (Owczarek et al. 2017).

Impairment of the pre-rRNA processing resulting in reduced ribosome biogenesis and cell divisions through ARF responding to the hyperproliferative signals seems to be related to inactivation of the pro-oncogenic role of NPM. It was proved that ARF interacted with NPM which facilitated polyubiquitination and then rapid proteasomal degradation of the latter. Moreover, NPM is induced by the Ras oncogene merely in the ARF-deprived cells which additionally proves ARF anticancer function (Itahana et al. 2003; Pandit and Gartel 2015). Besides, the activity of ARF itself depends on NPM, because its nucleolar localization is conditioned by the sequestration with NPM, provided by specific interaction between these two proteins (Luchinat et al. 2018), which limits its extranucleolar functioning (Korgaonkar et al. 2005). Moreover, it has been proposed that this interaction creates specific microenvironment facilitating nucleolar stress response (Mitrea and Kriwacki 2018). On the other hand, stabilization and level of ARF depend on its translocation to the nucleoplasm with aid of the glioma tumor suppressor candidate region 2 (GLTSCR2) protein (Lee et al. 2017a) and there on the ubiquitin–proteasome degradation mediated by the ARF ubiquitin ligase ULF/TRIP12 (Thyroid hormone Receptor Interacting Protein 12) (Chen et al. 2013). NPM can also act as a suppressor. In this role, NPM in a nucleolus interacts among others with Fbw7γ, a protein component of E3 ligase involved in ubiquitination and degradation of c-Myc. This allows maintaining the adequate level of c-Myc and, thus, diminishes cell transformation, whereas lack of NPM increases its level. However, NPM has no impact on the c-Myc mRNA level (Bonetti et al. 2008).

RPs, both RPSs and RPLs, after biosynthesis are either incorporated into ribosomes or participate in the negative regulation of activity of some oncogenes. RPs can also be involved in the latter process when they are released from ribosomes under unfavorable conditions such as serum starvation (Bhat et al. 2004) which causes ribosome disintegration and elicits ribosomal stress. For example, RPS14 inhibits transcriptional activity of c-Myc directly as well as indirectly through its co-activator TRRAP. This prevents c-Myc interaction with promoters of c-Myc-activated genes which reduces cell proliferation. Moreover, RPS14 participates in degradation of c-Myc mRNA by means of miRNA (Zhou et al. 2013). Likewise, RPL11 binds to c-Myc domain, MB II (Myc box II), which precludes TRRAP recruitment and inhibits the activation of H4 acetylation in the promoters of genes whose expression is regulated by c-Myc (Dai et al. 2007a). Moreover, in human cells, RPL11 binds to a specific domain of c-Myc mRNA which is responsible for its degradation via miRISC (miRNA-induced silencing complex) pathway. Here, RPL11 helps in the interaction of this domain with miRISC and, in consequence, significantly reduces the level and activity of c-Myc mRNA and the protein, respectively. This is also the case under the ribosomal stress (Challagundla et al. 2011). The RPL11-mediated degradation of c-Myc mRNA is additionally supported by RPL5. Both RPs guide miRISC towards c-Myc mRNA (Liao et al. 2014). A cross-talk between c-Myc, p53, and RPL11 was reported to play a role in pathophysiology of Diamond–Blackfan anemia. RPL11 deficiency is suggested to activate c-Myc-mediated synthesis of nucleolar proteins followed by triggering of p53-dependent response (Chakraborty et al. 2018).

Interestingly, functional interdependence between RPs and c-Myc allows for mutual regulation of activity of each of these factors which together can control ribosome biosynthesis. RPs influence the activity of c-Myc and the latter controls ribosome biogenesis i.a. through the transcription regulation of genes that encode RPs (Dai et al. 2007a; van Riggelen et al. 2010). Such cooperation was observed between RPL11 and c-Myc (Dai et al. 2007b). Participation of RPs in this mechanism translates into the control of cell growth and proliferation.

## A nucleolus as an opponent of cancer cells via p53-dependent pathways

In response to stress, including oncogenic one, some nucleolar factors participate in the induction of pathways that counteract the signals leading to hyperproliferation, and thus, these pathways prevent impaired or transformed cells from spreading. The change of a hitherto operating cell program into pathways leading cells to the defined destinations takes place. The activation of these pathways often occurs upon ribosome biosynthesis disturbance, i.e., upon nucleolar stress (Fig. 5).

Predominant and best-known pathways are those activated by p53 that regulates activity of many genes responsible for processes related to the cell life cycle (Menedez et al. 2009) both under normal and abnormal conditions. It was reported that a few hundred genes are differently expressed and a number of genes are downregulated in various tissues by p53 under irradiation stress (Tanikawa et al. 2017). Action of p53 is regulated by its cellular level. In normal cells, a low level of p53 is maintained by Mdm2, which directs p53 to the proteasomal degradation, whereas, under various stresses, including nucleolar one, p53 protects cells taking part in a stress response. Under such conditions, Mdm2 activity may be blocked by the nucleolar factors which results in the increased level of p53 and its stabilization in the nucleoplasm. Then, Mdm2-p53 pathways are activated to counteract the effects of stress, including oncogenic one. Certain proteins that are related to a nucleolus play an essential role in Mdm2 inactivation and, thus, in activation of these pathways. p53-mediated pathways lead to modifications of cell scenario such as cell cycle arrest, DNA repair, cellular metabolism modulation, apoptosis, or aging (Lazo 2017). As Mdm2 not only shows pro-oncogenic activity in the above-mentioned context, but it also plays a key role in tumor propagation and increase in metastatic potential when it is overexpressed (Venkatesan et al. 2018), and thus, it is another significant target in anticancer therapy (Carvajal et al. 2018; Zhou et al. 2018c).

It is thought that, in response to impairment of ribosome production, NPM plays an important role in the activation of pathways in which other nucleolar and ribosomal proteins besides p53 are involved. Most extracellular insults, including physical and chemical ones, result not only nucleolar stress but also in its oxidation. The change of redox status in a nucleolus leads to S-glutathionylation of NPM and releases it from a nucleolus to the nucleoplasm. It is probable that this typical NPM translocation during nucleolar stress is a prerequisite for p53 activation, so NPM is thought to be a molecule recognizing nucleolar stress. Therefore, nucleolar or ribosomal proteins alone are supposed to be insufficient to induce p53 pathways until NPM is translocated to nucleoplasm (Yang et al. 2016b). As such, a function of NPM has been discovered very recently; the reports do not mention common NPM participation in activation of the p-53-dependent pathways in response to nucleolar stress. Thus, only other nucleolar factors involved in the activation of appropriate pathways are taken into consideration. There is a growing body of evidence that RPs help to cope with unwelcome cells through their participation in pathways with or without p53 in response to the disruption of nucleolar function and integrity (Liu et al. 2017b; Russo and Russo 2017). Recent studies point to the fact that RPs exhibit certain regularity of action during either fight with or generation of cancers. Those RPs which mediate activation of the p53 pathways show negative deregulation in cancers and seem to be tumor suppressors, e.g., RPL5, RPL11, or RPL26, whilst other RPs, e.g., RPL36A or RPS2, are overexpressed in various cancers and are related to the increased cell proliferation; however, paralogs of these RPs can exert antagonistic effects as in the case of RPL26L1 (Guimaraes and Zavolan 2016).

Although NCL is known to be able to stabilize p53 (Saxena et al. 2006), another mechanism has been proposed, different from that with RPs, in which the conformational changes of human NCL cause exposure and availability of its specific domains capable of binding Mdm2. This blocks Mdm2 ligase activity which prevents ubiquitination and degradation of p53, thereby resulting in its activation (Bhatt et al. 2012).

The inhibition of casein kinase 1δ/ε (CK1δ/ε; yeast homologue Hrr25) activity results in lack of phosphorylation of Ltv1, a factor involved in the maturation of small ribosomal subunits (SRSs, 40Ss). This causes a permanent bond between this factor and pre-40Ss and prevents their maturation, and thus, the number of functional ribosomes decreases which leads to inhibition of cell proliferation and growth. Since CK1δ/ε overexpression is observed in several types of malignancies (Ghalei et al. 2015), development of its inhibitors might be a strategy to combat cancers, including breast cancer (Monastyrskyi et al. 2018).

Another nucleolar protein, cellular senescence-inhibited gene (CSIG, also known as ribosomal L1 domain containing 1, RSL1D1), is involved in processes such as cellular senescence and apoptosis. In addition, it regulates PTEN activity and is responsible for nucleolar localization of some proteins, including NS (Ma et al. 2008; Xie et al. 2016). Furthermore, its crucial role in regulation of Mdm2-p53 pathways was revealed. Due to that, under nucleolar stress, CSIG translocates from a nucleolus to the nucleoplasm where it connects through its ribosomal L1 domain with RING finger domain of Mdm2 and impairs its ligase activity. This stops ubiquitin-depended degradation of p53, stabilizes it, and activates appropriate pathways, while, when CSIG is knocked down, p53 activation is impaired which abrogates cell cycle arrest in G1 phase. Moreover, the block of Mdm2 RING finger domain probably prevents self-ubiquitination and degradation of Mdm2 (Xie et al. 2016).

1A6/DRIM (human UTP20) is a protein belonging to U3 proteins (t-UTPs) coordinating i.a. rDNA transcription through cooperation with UBF-1 and its acetyl-transferase hALP at rDNA promoter gene. The lack of UBF acetylation resulting from siRNA-mediated knockdown of 1A6/DRIM activity impairs initiation of RNA polymerase I-directed transcription and ribosome production. Then, ribosomal stress leads to activation of p53 pathway and to cell cycle arrest. On the other hand, 1A6/DRIM upregulation, promoting cell proliferation, occurs in certain cancer types (Peng et al. 2010).

The protein interacting with carboxyl terminus 1 (PICT-1) also known as the glioma tumor suppressor candidate region 2 (GLTSCR2) is a human nucleolar factor involved in ribosome biosynthesis, mainly in rDNA transcription and ribosomal subunits assembly. Disruption of PICT-1 functioning impairs ribosome production at various stages, thus leading to the activation of pathways directing cells towards concrete fates. PICT-1 is phosphorylated by ataxia–telangiectasia-mutated (ATM) kinase in a nucleolus which guides it to the proteasomal degradation in the nucleoplasm, among others in response to DNA damages. Decreased PICT-1 level elicits nucleolar stress followed by release of RPL11 from a nucleolus to the nucleoplasm, where RPL11 inhibits Mdm2 action and activates p53-mediated apoptotic pathway (Chen et al. 2016a). Interestingly, PICT-1 overexpression causes the reduction of UBF phosphorylation which considerably limits RNA polymerase I recruitment to r-gene promoters and inhibits rDNA transcription. This inactivates the AKT/mTOR/p70S6K signaling pathway and induces p53-independent autophagy in response to serum stimulation in human glioblastoma and breast adenocarcinoma cells. In addition, PICT-1 cooperates with tumor suppressor PTEN in the cytoplasm which causes anticancer effects. Given that PICT-1 mediates inhibition of cell proliferation and promotion of apoptosis and autophagy, while PICT-1 knockdown acts conversely and additionally enhances malignant progression of some cancers, PICT-1 itself can be considered as a suppressor (Chen et al. 2016b). Its suppressive role was additionally confirmed by the fact that disruption of PICT-1 expression was significantly correlated with pathogenesis and neoplastic progression of endometrial cancer (Yoshimoto et al. 2018). Therefore, PICT-1-dependent ribosome biosynthesis deregulation may be a strategy to fight cancer cells.

Among RPs, those which are involved in late-assembling of large ribosomal subunits, mainly to RPL11 and RPL5, are attributed the most important role in homeostasis and p53 stabilization. They together with 5S rRNA (5S ribonucleoprotein particle, 5S RNP, rRNA in complex with proteins) inhibit Mdm2 activity and maintain the structural integrity of nucleoli (Nicolas et al. 2016). 5S RNP–Mdm2–p53 pathway may be activated upon impairment of ribosome production, including downregulated RPS19 synthesis. Such a response significantly delays the initiation of AML, while it is not efficient when the disease is already established (Jaako et al. 2016).

Regulation of the activity of factors involved in start-up of particular pathways is complex, ambiguous, and can be carried out at several levels. It depends on their interaction with specific domains or with other factors as well as on a cell type and certain cellular settings, i.e., physiological condition of a cell, and impact of intracellular stimuli or of external environment. An exemplary situation occurs in human cells in the case of p53 regulation by two proteins related to a nucleolus, NCL and RPL26. In stress-free cells, NCL inhibits expression of p53 at translational level through binding to p53 mRNA, whereas under stress, NCL in combination with RPL26 facilitates translation of p53 which stabilizes it and p53-dependent pathways are consistently activated (Chen et al. 2012).

Based on the above-mentioned examples, it could be supposed that affecting nucleolar factors could be a potential mechanism eliminating defective cells which is generally beneficial for an organism in most cases (see below). However, ribosomal or nucleolar perturbations can promote cancer in certain situations. Such a situation is observed in colorectal cancer cells that first are subjected to the ribosome-inactivating stress (RIS) and next to anticancer agents. RIS imposes chemoresistance, i.e., it restrains death of cancer cells and increases their chance for survival following anticancer drug administration which ultimately impairs the efficiency of chemotherapy (Oh et al. 2016).

## Nucleolus-mediated anticancer strategies

Optimal functioning of cancer cells depends on intense protein biosynthesis. Since the main stages associated with formation of translational machineries, a process driving cell growth and proliferation as well as ensuring survival of every cell type, take place in a nucleolus, it accounts at least partly for carcinogenesis. Given that intense ribosome production favors transformation and cancer development, it means that cancer cells are susceptible to disruption of this process. Due to that, the intentional disruption of nucleolar functioning, which triggers nucleolar response followed by activation of appropriate pathways guiding damaged or dangerous cells, including transformed ones, to death, became an attractive anticancer strategy. Nucleolar dysfunction can be caused by the use of inhibitors, either in the form of chemical agents or RNAi, targeting any indispensable chain link at any stage of ribosome biogenesis, which would switch it off and trigger nucleolar stress. Therefore, every factor disturbing ribosome biogenesis and finally limiting cell growth and proliferation can be a potential tool in anticancer therapy (Fig. 2). For instance, targeted perturbation of RRP15 (ribosomal RNA processing protein) involved in nucleolar formation and ribosome biogenesis, including rDNA transcription and maturation of rRNA, interferes with nucleolar integrity and functioning and thereby induces nucleolar stress in human cells. This, depending on cellular p53 status, activates either RP–Mdm2–p53 pathway leading to the cell cycle arrest in p53-proficient non-transformed cells or ATR–ChkL–γH2Ax axis leading to the death of p53-deficient cancer cells (Dong et al. 2017).

There are many well-known synthetic and natural drugs targeting nucleolar functions which are used to combat cancer cells (Quin et al. 2014; Stępiński 2016; Woods et al. 2015). Interestingly, proteomic analyses revealed that upon exposure to nucleolar stress-eliciting antitumor drugs, regardless of the fact whether they originate from plants as TBMS1 (saponin from *Bolbostemma paniculatum*) (Lin et al. 2016b) or are obtained by chemical synthesis as oxaliplatin (Ozdian et al. 2017), proteins in cancer cells, including RPs, exhibit different expression, some are upregulated, and the others are downregulated. Different proteins whose expression changes during stress as well as those nucleolar or ribosomal proteins which are released from nucleoli as a result of stress-induced nucleolar disintegration may contribute to the activation of pathways eliminating undesired cells. The mechanisms associated with activation of both pathways dependent on (Vlatkovic et al. 2014) and independent of p53 (Chen et al. 2016a; Russo and Russo 2017) participate in the nucleolar surveillance response. Since more than half of human cancers are devoid of functional p53 (Soussi 2000), it is especially important to find the p53-independent anticancer pathways.

Most available classic chemotherapeutics, including those which selectively impair nucleolar functioning and trigger nucleolar stress response, are cytostatics that reduce or inhibit cell proliferation, and thus, intensively dividing cells, both cancer and normal ones, are targets for such drugs. Thereby, chemotherapy may impair functioning of certain tissues which leads to various side effects (Chorawala et al. 2012; Liu et al. 2015a). Therefore, on one hand, chemotherapy is beneficial, and on the other, it is a curse. Thus, as adverse effects often accompany chemotherapies, searching for strategies reducing them is very important.

CX-5461 is mainly used in malignances of hematological origin. It selectively inhibits rDNA transcription without nucleolar disintegration and thereby induces nucleolar stress and then p53-mediated apoptosis. As this drug specifically alters the structure of rDNA which mimics DNA damage and activates DNA damage response (DDR), it can also activate p53-independent pathways leading to modulations of cell cycle checkpoints via nucleolar ATM/ATR signaling (Quin et al. 2016). CX-5461 seems to be one of the less harmful drugs for normal cells. In vivo, it selectively drives mice premalignant and malignant B cells to apoptosis, while it is non-genotoxic for wild-type B-cell population. Analogous results were observed in cells originating from human hematologic cancers (Bywater et al. 2012). In addition, admittedly, CX-5461 was shown to be selectively toxic to acute lymphoblastic leukemia cells, without serious side effects on normal bone marrow cells; however, its application in combination with other agents such as specific inhibitors of ATM/ATR pathway allows for its lower doses, potentially reducing its cytotoxicity (Negi and Brown 2015). Thus, the novel CX-5461-induced nucleolar stress response is proposed to enhance therapeutic efficiency not only in p53-inefficient aggressive c-Myc-driven lymphomas but also in solid tumors through the combinational use of CX-5461 and ATM/ATR signaling inhibitors (Quin et al. 2016).

A new mechanism was revealed in which c-Myc-stimulated ribosome upregulation makes c-Myc-driven multiple myeloma cells more susceptible to nucleolar dysfunction. In this mechanism, CX-5461 elicits nucleolar stress and frees RPL5 from ribosomes, which mediates decrease in c-Myc level leading to the suppression of c-Myc-targeted gene expression. This inhibits growth and guides cells mainly to p53-independent apoptosis. In this case, the essential and novel possibility consist in the fact that antitumor action of CX-5461 can be applied not only in multiple myeloma but also in other c-Myc-driven cancers that additionally can be drug-resistant (Lee at al. 2017b). Interestingly, the use of CX-5461 to affect rDNA transcription and ribosome biogenesis not only in nucleolus but also in mitochondrion seems to be a possible strategy to combat c-Myc-driven cancers (Rossetti et al. 2018).

Targeting mTORC1 directly or indirectly is a promising anticancer approach (Mahoney et al. 2018), especially when mTORC1 signaling inhibitors are applied in combination with other drugs much better therapeutic efficacy is achieved. Temsirolimus, mTORC1 inhibitor, coupled with RG7388, an Mdm2 inhibitor, is effective therapy for p53-proficient neuroblastoma (Moreno-Smith et al. 2017). Devlin et al. (2016) evoked an interesting effect by application of the combined inhibition of rDNA transcription with CX-5461 and of PI3K-AKT-mTORC1-dependent ribosome and protein biosynthesis signaling with everolimus, mTORC1 inhibitor. In tests on mice, twice higher survival was achieved by the activation of two independent pathways triggering apoptotic tumor cell death, one with p53 and the other with BMF, a pro-apoptotic protein that was activated after the PI3K-AKT-mTORC1 pathway was inhibited. This seems to be a promising method to fight human c-Myc-driven B lymphoma (Devlin et al. 2016). 5-FU-resistant hepatocellular hepatoma become more sensitive to the drug when it operates in combination with mTORC1/2 inhibitors (Zhang et al. 2018). The examples quoted above prove that using at least two ways to disrupt the ribosome biosynthesis network can provide a more effective therapeutic tool to combat malignancies, including those in which c-Myc plays an essential role (Ni et al. 2018).

Human ribosome-free RPL3, accumulated as a result of its upregulation and release from ribosomes upon nucleolar stress, seems to be the critical participant in the response of lung and colon cancer cell lines lacking p53 to Act D, a transcription blocker that selectively inhibits rDNA transcription at low concentrations. Consistently, RPL3-mediated positive regulation of p21 expression via ERK (extracellular-signal-regulated kinase) activation results in promotion of apoptosis as well as reduction of proliferation and migration of these cancer cells (Russo et al. 2016a). In a similar experimental system, the use of 5-FU, DNA, and RNA synthesis inhibitor in combination with RPL3 overexpression considerably enhances sensitivity of 5-FU-resistant lung and colon cancer cells to this drug, and thereby, it strongly increases cytotoxic effect by means of the mitochondrial apoptotic cell response which prevents cell migration and invasion. Elicitation of such an effect involves RPL3-mediated negative regulation of two molecular targets in p53-null cells treated with 5-FU: (1) CBS (cystathionine-β-synthase), a factor enhancing energetics, proliferation, and migration of cancer cells and inflammation, and (2) NFҡB, a factor activated in many solid tumors linking cancer and inflammation. Since, in these cases, the success of therapy depends on RPL3 status; the treatment with Act D or 5-FU does not trigger the above-mentioned responses and makes drug chemotherapy ineffective when RPL3 expression is low. Thus, the development of agents increasing expression of this protein could make the fight with cancers more efficient (Pagliara et al. 2016; Russo et al. 2016b). Furthermore, RPL3 was reported to be an essential factor in acquiring multidrug resistance (MDR) of p53-deprived lung cancer cells through controlling the cellular redox status. In this scenario, 5-FU induces the resistance to multiple drugs that elicit nucleolar stress. RPL3 downregulation increases MDR, while restoration of its appropriate level re-sensitizes the cells resistant to nucleolar stress-inducing drugs by the regulation of oxidative stress responsive genes. Hence, maintenance of adequate RPL3 level seems to be crucial for anticancer therapeutic success, i.e., in response of p53-mutated lung cancer cells that acquired drug resistance (Russo et al. 2017). From a large number of RPs (33 of a small subunit and 47 of a large subunit), the involvement of RPL3 in activation of the anticancer pathways as a response to nucleolar stress induced by inhibitors of ribosome biogenesis seems to be well grounded in the case of p53-null cells as well as of those with acquired chemoresistance. 5-FU is commonly used in many solid tumors; however, it exerts cytotoxic effects by its active metabolite, 5-fluorodeoxyuridine monophosphate, not only in cancer but also in healthy cells causing numerous adverse effects (Thomas et al. 2016). Interestingly, polymorphic abnormality of genes responsible for 5-FU metabolism significantly enhances the toxicity of 5-FU-based therapy (Shahrokni et al. 2009a, b).

Platinum-derived agents, including oxaliplatin, are among anticancer chemicals targeting nucleolar function. Although oxaliplatin shows high antitumor cytotoxic activity, it is also responsible for adverse effects, mainly neurotoxicity (Argyriou 2015; Chukyo et al. 2018; Noh et al. 2018; Pereira et al. 2018). It is reported that oxaliplatin-derived neurotoxicity results from sodium channel dysfunctions which cause superexcitability of nerve axons (Heide et al. 2018). In addition, oxalate, a metabolite of oxaliplatin, is probably involved in the early development of peripheral sensory neuropathy and oxalate-induced toxicity might be mediated by ROS (Pereira et al. 2018).

Indeed, oxidative stress during which overproduction of ROS can modify cellular components, including DNA, is implicated in side effects accompanying chemotherapy. Dox, which affects ribosome biosynthesis, is one of the drugs with proved high pro-oxidant activity. Although it is an efficient anticancer agent with a broad spectrum of action from hematological cancers to solid tumors, its administration is restricted because of side effects. Cardiotoxicity is the major detriment upon application of Dox which significantly decreases antioxidant compounds in tissues (Dogan et al. 2014). Oxygen-derived free radicals damage nuclear and mitochondrial DNA which induces apoptosis of cardiomyocytes, thus antioxidative strategy should be introduced for cardioprotection (Mobaraki et al. 2017). It was reported that Dox and its metabolite, doxorubicinol, and impaired functioning of cardiac Ca^2+^ signaling pathway, because they bind to its essential protein components. This mechanism, in addition to oxidative stress, certainly contributes to fatal cardiotoxic side effects (Hanna et al. 2014).

Since the conventional chemotherapy is usually toxic to healthy cells, natural bioactive chemoprotective compounds with rare or negligible adverse effects are in the focus of interest. Oxaliplatin-induced neuropathic side effects could be mitigated by pre-treatment with AC591, a herbal medicine composed of certain neuroprotective herbs (Cheng et al. 2017; Noh et al. 2018). However, the combined treatment of Dox with a plant-originating antioxidant polyphenolic compound, cichoric acid, revealed that the latter reduced oxidative stress and apoptosis in human normal fibroblasts, so it can counteract Dox-induced cytotoxicity (Hajra et al. 2018; Jabłońska-Trypuć et al. 2018). Moreover, plant-derived small molecules such as indole-3-carbinol (Hajra et al. 2018), d-methionine (Lin et al. 2018a), or vivartana (Gnanasekaran et al. 2017) were reported to protect certain tissue types against specific chemotherapy side effects.

Natural components, administered alone or as complementation of standard anticancer agents, get more and more attractive in anticancer therapy because of their softer side effects. It was reported that plant-derived compounds such as sulforaphane, ursolic acid, and betulinic acid have cytostatic effect. These phytodrugs induce oxidant-based nucleolar stress in breast cancer both in cell lines from wild as well as mutated p53 types. Disruption of nucleolar function results in activation of pathways leading to p21-mediated inhibition of proliferation in both cell populations. Thus, plant drugs evoking ribotoxic stress may be new promising candidates in breast cancer therapy even in the cells deprived of functional p53 (Lewinska et al. 2017). Moreover, sulforaphane can influence epigenetic histone acetylation in breast cancer cells and thereby can restore specific gene expression which interferes with tumor growth (Gianfredi et al. 2017). It is also capable of eliminating pancreatic carcinoma cells via autophagy without toxic adverse events, but this activity involves participation of ROS (Naumann et al. 2011). In addition, a phytochemical, farnesiferol C (FC), reduces the levels of both c-Myc and RPL11, which co-regulate each other, as well as it attenuates the expression of survival genes leading to apoptosis in NSCLC. Furthermore, puromycin or Dox combined with FC enhance its cytotoxic effect (Jung et al. 2016).

Although it was said in the previous paragraph that NPM was likely to be commonly implicated in nucleolar stress response, interestingly, specific C-terminal mutation of NPM (NPMmut), which is present in ca. one-third of patients with AML, does not induce nucleolar stress. Furthermore, it prevents neither Act D-induced nucleolar stress nor activation of the p53-dependent and independent pathways leading cells to death by apoptosis or necrosis, respectively. Since NPM status is not so important in the case of leukemias, AML patients with NPMmut may be expected to have a better outcome of therapy, which is associated with cellular localization of NPMmut (Brodská et al. 2016). It is worth noting that Act D in combination with inhibitors of histone deacetylase significantly enhances therapeutic effects (Brodská et al. 2016), whereas combined with other standard chemotherapeutic agents little contributes to therapeutic efficiency of these agents (Parsons-Doherty et al. 2014). However, although Act D is often successful in treatment of a variety of cancers and it is relatively well tolerated, treatment with Act D is restricted mainly by its toxicity at higher doses, but its application at lower doses in combination with other drugs to synergize therapeutic efficacy allows to minimize its genotoxicity toward normal tissues (Choong et al. 2009).

Acrolein (Acr), as ubiquitous environmental contaminant also abundantly present in tobacco smoke, elicits dangerous diseases. Paradoxically, toxicity of Acr makes it a potential agent to be used for killing cancer cells regardless of p53 status. Acr preferably binds to nucleolar DNA causing oxidative damage of rDNA. This leads to nucleolar disintegration, thereby preventing rDNA transcription and ribosome biogenesis which induces RPL11-mediated apoptotic pathways as a response to nucleolar stress in human cancer cells with active and inactive p53 (Wang et al. 2016).

Interestingly, some nucleolar factors, including RPs, apart from being diagnostic parameters, can be useful as indicators of the cell response to an inhibitor of a given pathway, i.e., applied chemotherapy. For example, phosphorylation status of RPS6 changes in response to the agents targeting any component of RTK (receptor tyrosine kinase)/K-RAS downstream pathways in gastric cancer cells. In addition to RAF/MEK/ERK pathway, also the axis with mTORC1 is responsive to MEK inhibitors which results in their lowered activity and thereby in reduced cell proliferation. The reduced phosphorylation of RPS6 is observed in these cases. This feature could be used prior to treatment to select those gastric cancer patients who are sensitive to MEK inhibition (Hirashita et al. 2016).

## Conclusion

A growing number of new data concerning nucleoli make this nuclear suborganelle less and less mysterious. Just over 2 decades ago, the nucleolus was solely perceived as a ribosome factory. Since then, it has been shown to be a plurifunctional structure involved in many crucial cellular processes, many of which also concern cancer cells. On one hand, ribosome biosynthesis itself and a number of nucleolar factors drive transformation and cancer development; on the other hand, vulnerability of cells to impairment of ribosome biogenesis may help to fight cancers. Moreover, changes of nucleolar morphology and of many nucleolar factors give valuable information in diagnosis of several cancer types.

Participation of a rather small number of nucleolar and ribosomal proteins both in signaling pathways favoring carcinogenesis and in those that combat cancer cells has been confirmed so far. Attribution of new functions to proteins connected with nucleolus and ribosome biosynthesis whose roles are already known as well as functional decipherment of many new proteins that were identified to reside for longer or shorter time in nucleoli whose roles have not been specified yet, will undoubtedly take place in the near future.

Moreover, development and introduction of new molecular methods increasing expression of nucleolus-associated molecules activating known pathways and discovering new pathways and nucleolar factors or factors interacting with nucleolar ones participating in destroying undesired cells as well as searching for new inhibitors targeting pathways leading to transformation and cancer development which can enhance the effect of the agents whose effect was disappointing are the challenges facing cancer researchers. Furthermore, search for and application of ways and factors enhancing responsiveness of cancer cells that acquired resistance to a given drug could make chemotherapy more effective.

Moreover, most chemotherapeutic agents are usually toxic towards normal cells, causing side effects. Minimizing adverse effects considerably improves therapeutic efficacy and comfort of patients. That is why, it is very important to develop new approaches reducing undesired toxicity in non-malignant cells and to introduce them into the therapeutic course. Although the mechanisms of selective disruption of different stages of ribosome biosynthesis by different chemotherapeutics are well known, the exact mechanisms and factors contributing to selective elimination of cancer cells through drug-induced nucleolar stress which simultaneously do not harm healthy cells are not quite so well known yet. It was proved that intense ribosome biosynthesis, which is characteristic of many cancer types, is vulnerability of these cells that can be readily targeted. Indeed, cells with high rate of ribosome production are much more sensitive to perturbation of this process than those characterized by low rate of rRNA synthesis. Selective inhibitors of ribosome biosynthesis may cause cytotoxic effects leading to apoptosis of the cancer cells, while only cytostatic effects, i.e., transient cell cycle arrest of the healthy cells. As most cellular responses to inhibition of ribosome biosynthesis are mediated by p53, wild-type p53 and its high enough level during nucleolar stress in malignant cells is a prerequisite for the induction of apoptosis in them, whereas tumor cells with low rRNA synthesis rate should be additionally intervened with a drug that would rise p53 level enough to effectively address them toward death (Scala et al. 2016). Recently, rDNA has been recognized as a genome region of high sensitivity, especially in rapidly proliferating cells, and hence, inhibitors such as BMH-21, that may intercalate rDNA loci, selectively affect neoplastic cells but not normal ones (Wei et al. 2018). It is not clear which molecular factors, including signaling and receptor molecules, if any, can sense the changes of ribosome biosynthesis rates, thereby allowing discrimination between malignant and normal cell types on the basis of their response to perturbed ribosome production. It seems that non-tumor cells have a much higher threshold for activation and stabilization of p53 in response to nucleolar stress than cancer cells. Most probably, the higher sensitivity of malignant cells with high proliferative capacity to perturbed ribosome biosynthesis results from disregulated expression of both activating mutations of oncogenes, especially overexpression of c-Myc, and loss-of-function mutations in suppressors which might contribute to the loss of cell cycle checkpoints and ribosome biosynthesis control. Furthermore, some agents, in addition to target ribosome production, may also exert genotoxic effects in the form of damaged DNA. As signaling pathways controlling DNA reparation are often inefficient in proliferating cancer cells in contrast to normal ones, the former are much more sensitive to chemotherapy.

Cyclotherapy seems to be an interesting cytoprotective strategy which could use induction of p53-mediated nucleolar stress response in the form of reversible cell cycle arrest triggered by specific perturbation of ribosome biosynthesis at certain stages in conjunction with cytotoxic treatment. This method might be used for selective killing of intensely dividing cancer cells that have lost p53 in p53-independent manner and for sparing proliferating non-malignant p53-proficient cells that become temporarily non-dividing ones. In addition, if any mutation associated with nucleolar functioning was a reason of cell transformation and cancer progression, then the product of mutated gene could be a specific target of the most promising modern chemotherapy, and personalized therapy/molecularly targeted therapy.

Moreover, as malignant cells are characterized by deregulation of signal transduction and metabolic pathways, including those associated with cellular nutrition, the specific dietary regimen seems to be a promising strategy to promote differential effects in non-malignant and cancer cells. In this case, therapeutic effects are based mainly on the deficit of cellular energy which is utilized mainly for processes associated with intense proliferation of disregulated cancer cells. In addition to the fact that the cancer cells weakened by this deficit are notably sensitive to chemotherapeutic drugs, including ribosome biosynthesis-perturbing ones, oncogenes prevent the activation of stress resistance in them. Consequently, cancer cells are selectively killed, mainly by autophagy. On the contrary, normal starved cells halt cell cycle through insulin-like growth factor-1 and shift energy deficiency towards survival (Buono and Longo 2018; Cangemi et al. 2016; Lee et al. 2010). Although reports concerning therapeutic effects directly connecting diet and ribosome production are lacking, relationship between these two seems to be plausible. As ribosome biosynthesis is the most energy-consuming process in human cells, cancer cells with high ribosome production must generate sufficiently large amount of energy. Hence, it could be expected that insufficiency of energetic sources, especially glucose, caused by severe diet, could affect ribosome biosynthesis through energetic- and nutrient-related signaling pathways and activate pathways leading to the inhibition of growth and proliferation or to death of starved cells, especially malignant ones sensitive to perturbed ribosome biosynthesis. In general, different types of dietary approaches, including specific forms of fasting and starvation, were proved to greatly reduce chemotherapy-related adverse effects as well as selectively protect normal cells and simultaneously sensitize cancer cells to chemotherapy (Lee et al. 2012).

Summing up, disregulated signaling, resulting from altered genetic programs, connecting energetic metabolism, nutrient availability, cell growth, and proliferation, as well as ribosome and protein biosynthesis determines high sensitivity of cancer cells to appropriate chemotherapeutics in comparison to normal cells. Hence, detailed recognition of differences, including pathways and their players under various stress conditions, between cancer and normal cells would allow for more precise hit in targeted cells which would reduce or even eliminate side effects.
